# MicroRNA-492 expression promotes the progression of hepatic cancer by targeting PTEN

**DOI:** 10.1186/s12935-014-0095-7

**Published:** 2014-09-20

**Authors:** Jianxin Jiang, Yi Zhang, Chao Yu, Zhipeng Li, Yaozheng Pan, Chengyi Sun

**Affiliations:** Department of Biliary-Hepatic Surgery, Affiliated Hospital of Guiyang Medical College, 28 Guiyi Road, Guiyang, Guizhou 550001 China; Zhongnan Hospital of Wuhan University, Institute of Hepatobiliary Diseases of Wuhan University, Wuhan, Hubei 430071 China

**Keywords:** microRNA, Hepatocellular carcinoma, PTEN, AKT

## Abstract

**Background:**

Aberrant microRNA (miRNA) expression plays an essential role in the pathogenesis of Hepatocellular Carcinoma (HCC). However, specific involvement of miRNAs in HCC remains incompletely understood. The aim of this study was to explore the relevant microRNAs involved in the development of HCC.

**Methods:**

MicroRNA microarray was used to screen for the differentially expressed miRNAs in cancerous tissue and adjacent non-cancerous control tissue from patients with HCC (n = 3). Quantitative PCR was subsequently used to verify the results of microarray. Based on the findings, we investigated the role of miR-492 in the pathogenesis of HCC *in vitro* and *in vivo* using three tumor cells lines. Furthermore, we analyzed the clinical correlation of miR-492 expression with patient survival (n = 28).

**Results:**

We showed that microRNA-492 (miR-492) was elevated in HCC samples from patients with hepatic cancer. Knockdown of miR-492 attenuated the proliferation of cancer cell lines *in vitro* and inhibited primary tumor growth *in vivo* in SCID mice. We identified PTEN as a functional target for miR-492. Overexpression of miR-492 resulted in decreased PTEN expression and was associated with increased AKT activation in cancer cell lines. Moreover, miR-492-mediated increase of the proliferation of cancer cells was able to be suppressed by a PI3K inhibitor and an AKT inhibitor. The HCC patients with high miR-492/low PTEN had poorer survival.

**Conclusions:**

miR-492 is implicated in the regulation of HCC progression through PTEN and AKT pathway. The data suggest that miR-492 is a biomarker of HCC and a potential therapeutic target for hepatocellular carcinoma.

## Introduction

Hepatocellular carcinoma (HCC) is one of the most common cancers and the leading causes of cancer-associated death. Chronic inflammation and cirrhosis as a result of viral hepatitis B and C, fatty liver and alcohol abuse are usually considered the culprits of HCC which usually has a very poor prognosis. Clinically only a very limited number of patients are eligible for potentially curative treatment options such as surgical resection followed by orthotopic liver transplantation. Therefore, there is an urgent need to develop effective therapeutic approaches for the large number of HCC patients with advanced disease. For a long time, hepatocarcinogenesis has been considered to be the result of different genetic alterations that ultimately lead to malignant transformation. Nowadays, cancer development is no longer thought to be induced only by genetic and genomic alterations, but also should be considered as a result of epigenetic alterations.

MicroRNAs (miRNAs), an abundant class of endogenous, small, noncoding RNAs of ∼ 22 nucleotides in length, are post-transcriptional regulators that bind to specific cognate sequences in the 3′-untranslated region (3′-UTR) of target transcripts, usually resulting in translational repression and gene silencing. They are expressed in a tissue-specific manner and play an important role in cell proliferation, apoptosis, and differentiation. It has been shown that miRNAs play crucial roles in the regulation of diverse biological processes, such as development, inflammation, and tumorigenesis [1]. It has become evident that the expression level of miRs globally altered in cancer. MiR-21 has recently been found to be closely associated with the progression of pancreatic cancer. *In vivo*, miR-21 depletion stopped the progression of a very aggressive model of pancreatic ductal adenocarcinoma [2]. MiR-200 is a powerful marker and determining factor of the epithelial phenotype of cancer cells [3]. MiR-183 is dysregulated in breast cancer and its expression correlates with estrogen receptor and HER2/neu receptor expression. Induced overexpression of miR-183 inhibited migration of breast cancer cells [4]. It was recently found that miR-122 was downregulated in HCC. The oncogene c-Myc repressed miR-122 gene expression by associating with its promoter and by down-regulating Hnf-3β expression, whereas miR-122 indirectly inhibited c-Myc transcription by targeting Tfdp2 and E2f1 [5]. Furthermore, it was reported that miR-101, an important tumor-suppressive miRNA in human hepatocarcinomas, was epigenetically repressed by PRC2 complex in a c-Myc-mediated manner [6]. Therefore, micro RNA-mediated regulation plays a critical role in the development of hepatocarcinomas.

In this study, we investigated the possible involvement of microRNAs governing the expansion and functions of hepatic cells. We compared the expression of miRs in HCC specimens derived from patients with hepatic cancer and found that miR-492 is elevated in HCC. Further *in vitro* and *in vivo* experiments were performed to demonstrate the critical role of miR-492 in regulating cancer cell growth and promotion of tumor progression. We also investigated the mechanism which underlies the regulation of tumor cells by miR-492 and showed that miR-492 activated the PI3K/AKT pathway through targeting phosphatase and tensin homolog (PTEN). These findings provide new insights into the molecular mechanisms that regulate the expansion and functions of hepatic cancer cells and the exploration of potential targets for therapeutic intervention.

## Materials and methods

### Cell lines, animals and patient samples

HCT116, HuH-6 and HepG2 cells were cultured in Dulbecco’s Modified Eagle Medium supplemented with 10% fetal calf serum, L-glutamine, antibiotic and antimycotic cocktail (Life Technologies, USA). The cells were incubated at 37°C in a humidified atmosphere with 5% CO2. SCID mice were purchased from Shanghai Slac animal center (Shanghai, China). The animal protocol was approved by the institutional animal care committee of Guiyang Medical College, China. A total of 28 human primary liver cancer samples and their paired adjacent non-cancerous tissue samples were obtained with informed consent from patients of the Affiliated Hospital of Guiyang Medical College, China. Clear hepatocellular carcinoma was diagnosed histopathologically. Of these samples, two-third of patients were with cirrhosis. Twenty-two percent of patients were positive for HbsAg and Seventeen percent of patients were positive for anti-hepatitis C (HCV) antibody. The tumor sizes of 8 patients were less than 2 cm while that of 13 patients were between 2 cm and 3 cm. The tumor sizes of 7 patients were greater than 3 cm.

### Microarray assay

Total RNA was isolated using Trizol reagent (Invitrogen) and RNeasy Mini Kit (Qiagen), and the samples were then labeled using the miRCURY Hy3/Hy5 Power labeling kit and hybridized on the miRCURY LNA™ Array (v.11.0). Scanning was performed with the Axon GenePix 4000B microarray scanner. The intensity of green signal was calculated after background subtraction, and replicated spots on the same slide were averaged by getting a median intensity. We used the median normalization method to obtain “normalized data”: normalized data = (foreground-background)/median; the median represents the 50% quantile of miRNA intensity which is >50 in all samples after background correction. Significance of results was determined via fold change and *t* test. The threshold value we used to screen differentially expressed miRNAs was fold change > =1.5 or fold change = <0.8 and *p* value <0.05.

### Transfection of siRNA

Cells were transfected with Lipofectamine RNAiMax reagent (Invitrogen) according to the manufacturer’s guidelines. In brief, at the day 6 of culture, 5 × 10^5^ cells were plated in antibiotic-free culture medium and transfected with 100 *p*mol/well siRNAs. After overnight incubation, the medium was supplemented with antibiotics and the cells were used for further experiments.

### Gene expression analysis

Total RNA was isolated from cell lines with Trizol reagent (Invitrogen) according to manufacturer’s instructions and the RNA concentration was measured with the ND-1000 NanoDrop spectrophotometer (Nanodrop). miRNAs were quantified from 1 μg total RNA using the miScript PCR System (Qiagen). U6 and 5S RNAs were used as internal controls. cDNA samples were diluted 1 into 100 for miRNA detection or U6 and 1 into 10,000 for 5S RNA detection.

### Autologous xenograft

Recombinant HIV-1-derived lentiviral vector was produced following transfection of HEK293 T cells (2 × 10^5^) with plasmids using per 10 cm dish: 5 ug of pLenti-shRNA construct, 6 ug of packaging plasmids using MegaTran transfection reagent with Opti-MEM16 according to manufacturer’s instructions (Origen, China). Medium was changed the day following transfection and collected 24 hours later to harvest the produced viral particles which were purified by centrifugation. Lentiviral vectors were titered by physical titers measuring P24 and reverse transcriptase (RT) levels by ELISA. There were 10 SCID mice (5 in each group) that received an injection in the right flank using 5 × 10^6^ of HCT116 cell line infected with lentiviral scramble or anti-miR-492 (day 0). Tumors were found in all these mice and were palpable 14 d later. On day 30, all mice were sacrificed and the tumors were isolated for evaluation of the volume (volume = length × width2 × π/6).

### Colony formation

2 × 10^3^ resuspended cells were placed in methylcellulose media and the vial was vigorously vortexed to thoroughly mix cells with the media. After approximately 20 minutes, 1.1 mL of the final cell mixture was added to a 35 mm culture plate using a 3 mL syringe fitted with a 16 gauge needle with gently rotating the plate to allow the media spread evenly. Two sample plates and an uncovered plate containing 3–4 mL sterile water were placed in a 100 mm culture plate with cover. The sterile water plate served to maintain the humidity necessary for colony development. The cells were incubated for 5–10 days at 37°C and 5% CO_2_ without disturbing the plates during the incubation period to prevent shifting of the colonies. Individual colonies representing cell growth were counted as previously described [7].

### 3′-UTR luciferase reporter assay

The PTEN 3′-UTR luciferase reporter construct was made by amplifying the PTEN mRNA 3′-UTR sequence and cloning it downstream of the CMV-driven firefly luciferase cassette in the pMIR-REPORT vector (Ambion). HEK-293 cells were co-transfected with 80 ng luciferase reporter plasmid, 40 ng pRL-TK-Renilla-luciferase plasmid, and miR-492 (final concentration, 20 nM). Luciferase activities were measured using the Dual-Luciferase Reporter Assay System (Promega).

### MTT cell proliferation assay

Briefly, cells were seeded at 5 × 10^3^ cells per well in a 96-well dish and grown in 10% FCS DMEM medium. After 24 h, cells were infected with lentivirus (miR-492 or control). The proliferation of cells was determined using MTT Cell Proliferation Assay (Promega) according to manufacturer’s instructions at different time points following infection.

### Western blotting

Proteins were extracted from cell lines, resolved by SDS-PAGE and transferred to PVDF membrane. After blocking for 1 h at room temperature, blots were incubated overnight at 4°C with antibodies to PTEN, P-AKT, T-AKT, β-actin (Cell Signaling Technology) diluted according to the manufacturer’s recommendations. After wash, secondary HRP-conjugated antibodies (dilution 1:10,000; Pierce) were added and blots were incubated at room temperature for 1 h. The protein bands were visualized using ECL chemiluminescence (Pierce).

### Statistical analysis

Numerical data were expressed as mean ± SD. ANOVA and Student’s t tests were done to determine the differences in the means among the various treatment groups. *p* <0.05 was considered statistically significant. The SPSS 10.0.2 software package (SPSS, Inc.) was used for analysis. The Kaplan-Meier survival curve was plotted with Graphpad Prism 5.0 software (Graphpad Software, Inc.).

## Results

### miR-492 is elevated in HCC tissues from patients

**Figure 1:**
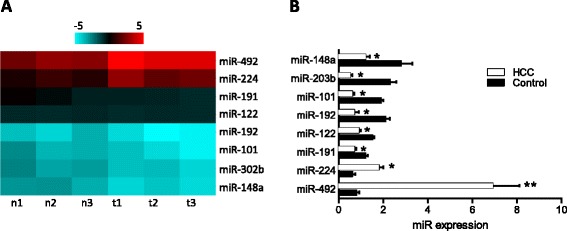
***Comparison of miRNA expression profile between HCC tissues and adjacent non-cancerous tissues using an array-based miRNA profiling.*** **(A)** The heat map diagram shows the result of the differentially expressed miRNAs (only differentially expressed miRNAs with fold change > =1.5 or fold change = <0.8 and *p* value <0.05 are included) from miRNA microarray. **(B)** The expression levels of these miRNAs were measured by qRT-PCR and normalized to the expression of U6 in each sample. Data are the mean ± SD (n = 3) of one representative experiment. Similar results were obtained in three independent experiments. n: adjacent non-cancerous tissues, t: HCC tissues. *refers to *p* < 0.05 and **refers to *p* < 0.01 in a Student’s *t* test between the HCC group and the control group.

By comparing the miRNA expression profile between HCC tissues and adjacent non-cancerous tissues using an array-based miRNA profiling, eight differentially expressed miRNAs were identified. To select the altered miRNAs, the cutoff criteria were set as that the ratio of miRNA by HCC to control was greater than 1.5 folds or less than 0.8 folds for upregulation or downregulation. Among all selected miRNAs, we choose the most upregulated and downregulated miRNAs for further analysis. Two were upregulated (miR-492 and miR-224) and six were downregulated (miR-191, miR-122, miR-192, miR-101, miR-302b, miR-148a) (Figure 1A). We confirmed the expression of these miRNAs by qRT-PCR analysis (Figure 1B). Consistent with the array data, the results showed that miR-492 expression is generally the highest and differs most significantly (tumor vs. control, *p* < 0.01).

### Knockdown of miR-492 inhibits cancer cell proliferation *in vitro*

**Figure 2:**
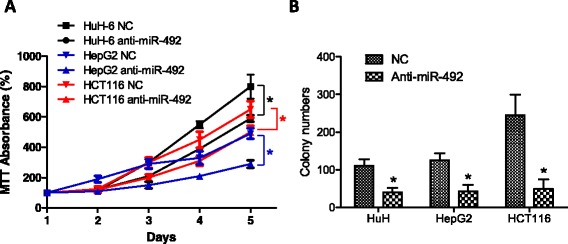
***Knockdown of miR-492 attenuates cencer cell proliferation and colony formation in vitro.*** **(A)** The expression of miR-492 was inhibited using siRNA transfection to cancer cell lines HuH-6, HCT116 and HepG2. Subsequently, the proliferations of cancer cells were determined using MTT. Statistical analysis was carried out on day 4 and day 5. **(B)** Colony formation of three cell lines were measured in the culture treated with siRNA to miR-492 and control siRNA (NC). Data are presented as mean ± SD. *refers to *p* < 0.05 between the groups.

To understand the functional relevance of miR-492 overexpression in cancer, the expression of miR-492 was downregulated by transfection of siRNA to miR-492 in human cancer cell lines HCT116, HepG2, and HuH-6. As shown in Figure 2A, knockdown of miR-492 significantly inhibited the proliferation of these cells (*p* < 0.05). Moreover, colony formation of these cell lines was also significantly inhibited by knockdown of miR-492 (Figure 2B).

### Knockdown of miR-492 attenuates tumor growth *in vivo*

**Figure 3:**
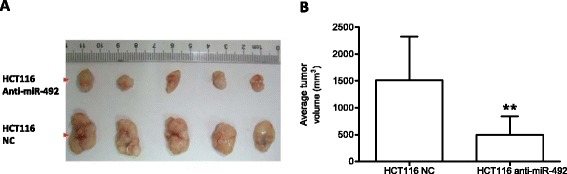
***Anti-miR-492 inhibits the in vivo growth of HCT116 in SCID mice*** **. (A)** HCT116 cells (5 x 10^6^) were infected with lentiviral siRNAs to miR-492 or scramble control and subsequently transferred into SCID mice (n = 5 per group). After 30 days, tumors were isolated from mice and their volume were calculated as “volume = length × width2 × π/6”. **(B)** The average tumor volume was calculated from 5 mice per group and statistically analyzed to compare the difference between the experimental group and the control group. **refers to *p* < 0.01.

We next examined the effect of miR-492 knockdown on tumor growth *in vivo* using orthotopic implantation of HCT116 cells infected with lentiviral scramble or anti-miR-492. After injection of equal amount cells into SCID mice for 4 weeks, autopsy was conducted to compare the tumor size. As shown in Figure 3, significantly smaller tumors were observed in the SCID mice injected with the HCT116 cells infected with the lentiviral anti-miR-492 compared to that in control animals (1507 ± 817 vs. 495 ± 342 mm^3^).

### PTEN is a functional target of miR-492

**Figure 4:**
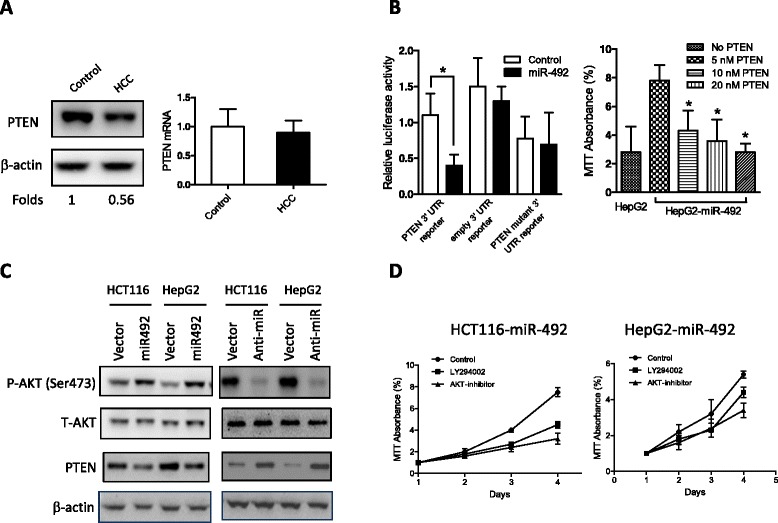
***PTEN is a functional target of miR-492 in HCCs*** **. (A)** HCC tissues and the adjacent non-cancerous tissues were subject to Western blot and real-time PCR analyses to assess PTEN mRNA and protein levels. Data are the mean ± SD (n = 4) of one representative experiment. Similar results were obtained in at least three independent experiments. **(B)** HEK-293 cells were transfected with either miR-492 mimics (20 nM) or the mutant 3′-UTR of miR-492 (20 nM) along with the matched control using 80 ng of indicated vectors and 40 ng of pRL-TK. After 24 h, firefly luciferase activity was measured and normalized by Renilla luciferase activity. Data are the mean ± SD (n = 4) of one representative experiment. Similar results were obtained in at least three independent experiments. The proliferation of HepG2 cells transfected with miR-492 plasmid (0.5 nM) and different concentrations of PTEN plasmid (5 nM, 10 nM, 20 nM) was determined by MTT. *indicates the significant difference between the control and PTEN transfected cells. **(C)** HCT116 or HepG2 cells were infected with the indicated lentivirus for 24 h, and p-AKT, total AKT and PTEN levels were detected by immunoblot analysis. Data shown is one representative experiment out of three. **(D)** miR-492 transfection enhanced the proliferation of both HCT116 and HepG2 which was inhibited by the PI3K and ATK inhibitors.

It is generally accepted that miRNAs exert their function through regulating the expression of their target genes. Thus, we took advantage of multiple prediction algorithms (miRBase, PicTar, and TargetScan v.5.1) to identify the potential miR-492 targets related to the functions of tumor-associated myeloid-derived suppressor cells (MDSCs). Among hundreds of potential target genes, PTEN, a major negative regulator of the PI3K/AKT signaling pathway involved in cell growth, apoptosis, motility, and differentiation, was demonstrated in this study to be a functional target of miR-492 in HCCs. We first compared PTEN mRNA and protein expression levels between tumor and adjacent non-tumorous tissues. Notably, the PTEN protein level was remarkably downregulated in tumor HCC tissues compared with the control whereas PTEN mRNA expression showed no difference (Figure 4A). The inconsistency between PTEN mRNA and protein levels hints that a miRNA-mediated mechanism may be involved. The regulation of PTEN by miR-492 ought to be investigated by the observation of the expression of PTEN protein but not the expression of PTEN mRNA. To confirm the possibility that PTEN may be regulated post-transcriptionally by miR-492, 3′-UTR of PTEN reporter plasmid was constructed. When the reporter plasmids with miR-492 mimics or the scrambled oligonucleotide were transfected into HEK-293 cells, we observed that the miR-492 mimics markedly decreased the luciferase activity (Figure 4B). To further confirm PTEN is a target of miR-492, we transfected PTEN plasmid to HepG2 cells to analyze the proliferation of HepG2 cells. The results showed that transfection of PTEN plasmid counteracted the effects of miR-492 by downregulating of the proliferation of HepG2 cells (Figure 4B). In HCT116 or HepG2 cells, overexpression of miR-492 resulted in the reduction of PTEN level, associated with increased AKT phosphorylation, whereas knockdown of miR-492 produced opposite effects (Figure 4C). Furthermore, miR-492-induced increases of proliferation of HCT116 and HepG2 were markedly reversed by PI3K and AKT inhibitors (Figure 4D).

### miR-492-PTEN is a clinically relevant pathway in HCC

**Figure 5:**
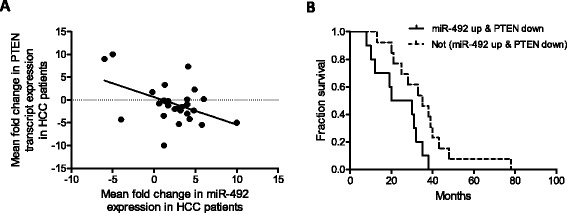
***Upregulation of miR-492 and downregulation of PTEN expression is associated with poorer survival of HCC patients*** **. (A)** Scatter plot represents the statistically significant correlation in the relative transcript expression of miR-492 and PTEN (*p* < 0.001) in the tumor versus paired adjacent non-tumor tissues from 28 HCC patients. Each spot represents data from one HCC patient presented in the Log2 scale and the linear regression line is marked as the solid line. **(B)** Kaplan-Meier survival curve for HCC patients classified based on whether HCC tumor showed miR-492 upregulation (more than 2 fold) and PTEN downregulation (more than 1.5 fold), compared to the paired adjacent non-tumor samples. Upregulation of miR-492 and downregulation of PTEN expression is significantly associated with poorer patient survival (*p* <0.05).

We proceeded to evaluate the clinical relevance of our experimental observation by examining the expression of miR-492 and PTEN in tumor and paired adjacent non-tumorous tissues from 28 HCC patients. A significant inverse correlation (*p* < 0.001, r = −0.45, R2 = 0.122) was observed between the expression of miR-492 and PTEN in these HCC samples (Figure 5A), suggesting PTEN as a clinically relevant miR-492 target in HCC. Moreover, HCC patients with tumors which exhibits more than 2-fold upregulated miR-492 and more than 1.5-fold downregulated PTEN expression compared to the paired adjacent non-tumorous tissues were found to be significantly associated with poorer overall survival (*p* < 0.05, Figure 5B). Collectively, these data suggest that deregulation of miR-492 expression with subsequent deregulation of PTEN expression may serve as a prognostic biomarker predicting the patients’ survival outlook.

## Discussion

Although extensive efforts have been devoted to understanding the molecular pathogenesis of HCC, few effective treatments currently exist. Systemic chemotherapy and radiotherapy, two conventional cancer therapies, exhibited low response rates and no demonstrated survival benefits in HCC [8]. Treatment with Sorafenib, an approved multikinase inhibitor, resulted in only modestly prolonged survival [9]. Development of novel therapeutics for the prevention and treatment of HCC is therefore urgently needed. Despite the general lack of activity of conventional therapeutics in HCC, hepatocytes have been shown to readily take up antisense oligonucleotides [10], raising the hope that small nucleic acid-based molecules may have utility against HCC. MicroRNAs (miRNAs) are small, endogenous RNAs that regulate gene expression through messenger RNA (mRNA) degradation or inhibition of translation [11]. Aberrant miRNA expression plays an essential role in HCC pathogenesis, and miRNA replacement or inhibition therapies have been suggested as potential therapeutics for HCC [12]. As proof of principle, recombinant adeno-associated virus (AAV)-mediated overexpression of miR-26a was recently shown to attenuate primary MYC-driven tumor formation in a transgenic (Tg) mouse model [13]. Despite the safety concerns on using adenovirus, the development of new therapies based on miRNA manipulation is a current research focus and believed to be the next generation of therapy for various cancers.

Not only deregulated gene expression but also the alteration of post-transcriptional gene silencing mediated by microRNA were demonstrated to influence pathogenesis of human cancers by either acting as tumor suppressor or as oncogene [14]. MiRNAs originated from introns of protein and nonprotein coding genes or even rarely from exons [15]. Of note, distinct miRNA signatures have already been used for the classification and prognosis of various cancers, including HCC [16-18]. Previous studies showed both consistent and inconsistent results regarding the correlation between miR-492 and the development of gastrointestinal cancers. It was reported that miR-492 could originate from the coding sequence of the hepatoblastoma marker gene keratin 19 (KRT19) and miR-492 along with its associated targets might serve as new hepatoblastoma biomarkers of clinical utility and could assist to explore targeted therapies, especially in metastatic hepatoblastoma with a poor prognosis [19]. However, there was an inconsistent report describing that the gene polymorphisms of miR-27a, miR-146a, miR-196a-2, miR-492, miR-492a and miR-608 were not associated with the presence of colon cancer in European subjects [20]. It should be noted the upregulation of miR-492 was not limited to a specific type of liver cancer as it was also considered a biomarker for hepatoblastoma besides HCC. In addition to miR-492, other miRs such as miR-494 and miR-449 were also reported to be associated with various cancers [21-23]. miR-145 potently suppressed growth of three different colon carcinoma cell line [24]. However, the exact functional role of miRNAs in the development, progression as well as its regulatory pathway remains unclear. Our findings suggested a novel miR-492-PTEN pathway implicated in the progression of HCC. It is conceivable that PTEN is not exclusively regulated by a specific miRNA; other miRNAs such as miR-494 likely regulates PTEN too. This brings up the question whether the increase of miR-492 is relevant to a particular cancer stage. In our study, the expressions of other miRNAs such as miR-224, miR-148a has been found to either increase or decrease in HCC. The role of those miRNAs in the pathogenesis of HCC might be worth exploration.

Given that PTEN acts as a major negative regulator of the PI3K/AKT signaling pathway, the repression of PTEN by miR-492 might play an important role in proliferation of other types of cell too. It was reported that the activity of MDSC was regulated by miR-494 through PTEN/AKT. The downregulation of PTEN by miR-494 enhanced the activity of AKT and its downstream pathways, promoted the accumulation of MDSCs in tumor tissues [23]. Thus, tissue specific regulation of PI3K/AKT pathway should also be addressed in this regard. In conclusion, this study described the upregulation of miR-492 in cancerous tissue of hepatocellular carcinoma and depicted a new miR-492-PTEN-AKT pathway in the progression of hepatocellular carcinoma. It also showed that miR-492 upregulation/PTEN downregulation correlated with the survival outlook of patients with HCC.
